# Efficacy of acyclovir for herpes simplex encephalitis: A protocol for a systematic review of randomized controlled trial

**DOI:** 10.1097/MD.0000000000015254

**Published:** 2019-04-12

**Authors:** Wei Wang, Meng Ji

**Affiliations:** Department of Neurolgy, Beijing Chaoyang Hospital, Capital Medical University, Beijing, China.

**Keywords:** acyclovir, efficacy, herpes simplex encephalitis, randomized controlled trial, safety, systematic review

## Abstract

**Background:**

Clinical researches indicate that acyclovir can be used to herpes simplex encephalitis (HSE). However, no systematic review has explored its efficacy for the treatment of HSE. Therefore, this study systematically will investigate the efficacy and safety of acyclovir for patients with HSE.

**Methods:**

We will search the following databases from inceptions to March 1, 2019 without any language restrictions: Cochrane Library, Embase, MEDICINE, PsycINFO, Web of Science, Allied and Complementary Medicine Database, Chinese Biomedical Literature Database, and China National Knowledge Infrastructure. This study will include randomized controlled trials that assess the efficacy and safety of acyclovir for patients with HSE. Two authors will independently carry out the study selection, data extraction, and risk of bias assessment. Cochrane risk of bias tool will be used to assess the risk of bias assessment.

**Results:**

This study will systematically assess the efficacy and safety of acyclovir for HSE. The primary outcome is mortality rate, which is measured by Glasgow coma score, or other instruments. The secondary outcomes include quality of life, as assessed by 36-Item Short Form Health Survey or relevant scales; overall survival, the number of patient who died; the number of patient who had severe sequelae, and adverse events.

**Conclusions:**

The findings of this study may provide the existing evidence on the efficacy and safety of acyclovir for HSE.

**PROSPERO registration number:**

PROSPERO CRD42019125999.

## 1 Introduction

Herpes simplex encephalitis (HSE) is one the most common factors that can result in sporadic focal encephalitis worldwide.^[1–3]^ It mainly manifests with fever, convulsions, confusion, focal neurologic signs and progressive deterioration.^[4–6]^ Normally, it is diagnosed according to the results of herpes simplex virus detection in the cerebrospinal fluid.^[7]^ It is estimated that the incidence of HSE ranges from 1 to 2 cases per 500,000 population each year.^[8]^ Its mortality and morbidity rates are very high around 70% without effective antiviral therapy. It is still high even the patients are treated with antiviral therapy.^[9]^

A numerous clinical studies have reported that acyclovir has been widely used for the treatment of HSE.^[10–25]^ However, to our best knowledge, no systematic review has assessed the efficacy and safety of acyclovir for patients with HSE. Thus, this study will firstly and systematically evaluate the efficacy and safety of acyclovir for patients with HSE.

## 2 Methods and analysis

### 2.1 Study registration

This study has been registered on PROSPERO (CRD42019125999), and it has been reported abiding to the guidelines of Preferred Reporting Items for Systematic Reviews and Meta-Analysis Protocol statement.^[26]^

### 2.2 Eligibility criteria

#### 2.2.1 Types of studies

This study will only include randomized controlled trials (RCTs) of acyclovir for HSE. However, the studies belonging to the nonclinical studies, noncontrolled trials, and non-RCT will be excluded in this study.

#### 2.2.2 Types of interventions

The experimental intervention includes acyclovir monotherapy. However, acyclovir plus other treatments will be excluded. The control treatment can be any treatments except acyclovir.

#### 2.2.3 Types of participants

Patients with HSE will be included without any restrictions of race, sex, and age.

### 2.3 Types of outcome measurements

#### 2.3.1 Primary outcome

Mortality rate, as measured by Glasgow coma score, or other instruments.

#### 2.3.2 Secondary outcome

Quality of life, as assessed by 36-Item Short Form Health Survey or relevant scales; Overall survival;

Number of patient who died;

Number of patient who had severe sequelae;

Adverse effects (any expected and unexpected adverse reactions or effects).

### 2.4 Search strategy

#### 2.4.1 Electronic databases search

We will search the following electronic databases for relevant studies from inceptions to March 1, 2019 without any language restrictions: Cochrane Library, Embase, MEDICINE, PsycINFO, Web of Science, Allied and Complementary Medicine Database, Chinese Biomedical Literature Database, and China National Knowledge Infrastructure. In addition, we will also search reference lists of relevant studies. This study will only consider RCTs evaluating the efficacy and safety of acyclovir for HSE. The detailed search strategy for Cochrane Library is presented in Table 1. Identical search strategies for all other electronic databases will also be built and applied.

**Table 1:**
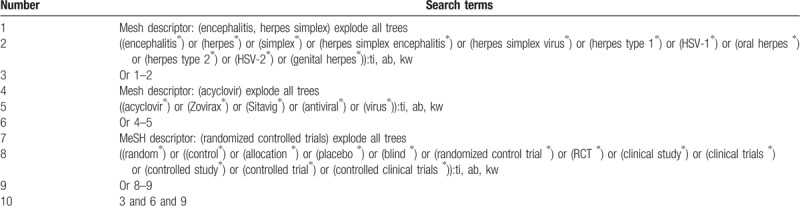
Search strategy sample of Cochrane Library.

#### 2.4.2 Other literature sources search

Other literature sources will also be searched, such as reference lists of relevant included RCTs and associated conference proceedings to avoid missing any potential studies.

### 2.5 Study selection

**Figure 1:**
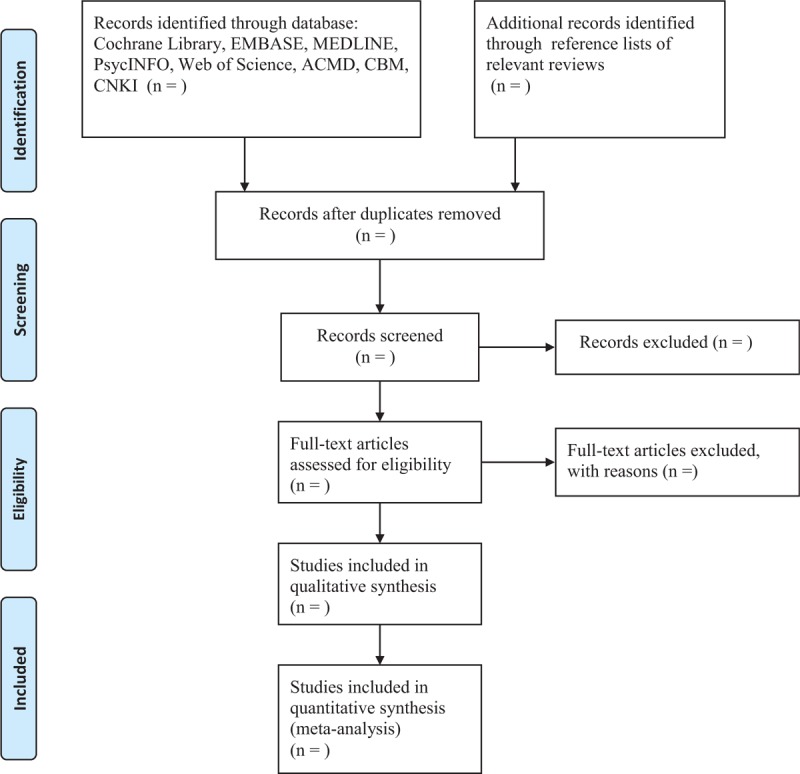
Process of study selection.

Two authors will independently conduct study selection by scanning titles and abstracts. Full text will be subsequently screened for finally selection based on the predefined eligibility criteria. Any disagreements regarding the study selection between the 2 authors will be solved by a third author through discussion. The results of whole procedure of study selection will be presented in Figure 1.

### 2.6 Data extraction and management

All data will be managed by using Endnote 7.0 software. Two authors will independently extract the following information. Any disagreements regarding the data extraction will be solved by consulting a third author.

General information: title, first author, year of study, location, and journal;

Patient information: race, age, diagnostic criteria, eligibility criteria, and number of patients.

Study methods: details of randomization, concealment, blinding, and other risk of bias.

Treatment details: intervention names, dosage, frequency, and duration.

Outcome measurement: all primary, secondary, and safety.

### 2.7 Missing data dealing with

Any missing data will be inquired the original authors by using email. If we cannot receive those data, only available data will be analyzed. Moreover, we will also discuss its possible affects in the text.

### 2.8 Risk of bias assessment

Two authors will independently evaluate the methodological quality for all eligible RCTs in this study by using Cochrane risk of bias tool. This tool comprises of 7 aspects. Each item will be judged as a high, or unclear, or low risk of bias. Any divergences regarding the risk of bias assessment between 2 authors will be resolved by a third author through discussion.

### 2.9 Statistical analysis

RevMan 5.3 software will be used to pool the data and perform meta-analysis if it is possible. All continuous values will be presented as mean difference and 95% confidence intervals. All the dichotomous values will be reported as risk ratio and 95% confidence intervals.

Heterogeneity among included studies will be identified by *I*^2^ test. If *I*^2^ ≤50%, minor heterogeneity will be considered. Then, data will be pooled by using a fixed-effect model, and meta-analysis will be performed. Otherwise, if *I*^2^ > 50%, a significant heterogeneity will be considered. Then, data will be synthesized by using a random-effect model. Meanwhile, subgroup will be conducted. Meta-analysis will be conducted according to the results of subgroup analysis. If there is still significant heterogeneity will be identified after the subgroup analysis, data will not be pooled, and meta-analysis will not be performed. However, a narrative description will be reported instead.

Additionally, subgroup analysis will be conducted to detect any potential reasons that may cause significant heterogeneity. It will be performed according to the different characteristics, treatment schedules, and outcome measurements. Sensitivity analysis will also be carried out to check the robustness and stability of combined results by removing low quality studies. We will also plan to operate the funnel plot and Egger's regression test if sufficient studies will be included in order to test the potential reporting bias.^[27,28]^

## 3 Discussion

Numerous clinical trials have hypothesized that acyclovir plays a very important role in the treatment of patients with HSE. However, no systematic review has reported the efficacy and safety of acyclovir for HSE, and thus it is still at the conceptual level. Considering numerous literatures on acyclovir for HSE,^[10–25]^ we will conduct a systematic review to inform the efficacy and safety of acyclovir for patients with HSE. The findings of the present study are expected to summarize the latest evidence regarding the efficacy and safety of acyclovir for HSE. In addition, the results of this study may also provide important evidence for both clinical practice and patients.

## Author contributions

**Conceptualization:** Wei Wang, Meng Ji.

**Data curation:** Wei Wang, Meng Ji.

**Formal analysis:** Meng Ji.

**Funding acquisition:** Wei Wang.

**Investigation:** Wei Wang.

**Methodology:** Meng Ji.

**Project administration:** Wei Wang.

**Resources:** Wei Wang, Meng Ji.

**Software:** Meng Ji.

**Supervision:** Wei Wang.

**Validation:** Wei Wang, Meng Ji.

**Visualization:** Wei Wang, Meng Ji.

**Writing – original draft:** Wei Wang, Meng Ji.

**Writing – review & editing:** Wei Wang, Meng Ji.
